# Anti-Epileptic Drug Combination Efficacy in an *In Vitro* Seizure Model – Phenytoin and Valproate, Lamotrigine and Valproate

**DOI:** 10.1371/journal.pone.0169974

**Published:** 2017-01-11

**Authors:** Kim Det Taing, Terence J. O’Brien, David A. Williams, Chris R. French

**Affiliations:** Department of Medicine (Royal Melbourne Hospital), The University of Melbourne, Melbourne, Victoria, Australia; University of Modena and Reggio Emilia, ITALY

## Abstract

In this study, we investigated the relative efficacy of different classes of commonly used anti-epileptic drugs (AEDs) with different mechanisms of action, individually and in combination, to suppress epileptiform discharges in an *in vitro* model. Extracellular field potential were recorded in 450 μm thick transverse hippocampal slices prepared from juvenile Wistar rats, in which “epileptiform discharges” (ED’s) were produced with a high-K^+^ (8.5 mM) bicarbonate-buffered saline solution. Single and dual recordings in stratum pyramidale of CA1 and CA3 regions were performed with 3–5 MΩ glass microelectrodes. All drugs—lamotrigine (LTG), phenytoin (PHT) and valproate (VPA)—were applied to the slice by superfusion at a rate of 2 ml/min at 32°C. Effects upon frequency of ED’s were assessed for LTG, PHT and VPA applied at different concentrations, in isolation and in combination. We demonstrated that high-K^+^ induced ED frequency was reversibly reduced by LTG, PHT and VPA, at concentrations corresponding to human therapeutic blood plasma concentrations. With a protocol using several applications of drugs to the same slice, PHT and VPA in combination displayed additivity of effect with 50μM PHT and 350μM VPA reducing SLD frequency by 44% and 24% individually (n = 19), and together reducing SLD frequency by 66% (n = 19). 20μM LTG reduced SLD frequency by 32% and 350μM VPA by 16% (n = 18). However, in combination there was a supra-linear suppression of ED’s of 64% (n = 18). In another independent set of experiments, similar results of drug combination responses were also found. In conclusion, a combination of conventional AEDs with different mechanisms of action, PHT and VPA, displayed linear additivity of effect on epileptiform activity. More intriguingly, a combination of LTG and VPA considered particularly efficacious clinically showed a supra-additive suppression of ED’s. This approach may be useful as an *in vitro* platform for assessing drug combination efficacy.

## Introduction

Despite the proven efficacy of many antiepileptic drugs (AEDs), about 30% of patients continue to have seizures, that is, are “drug resistant” [1–3]. Many patients with drug resistant epilepsy are treated with more than one AED, and combinations of drugs with different mechanisms of action are commonly used with enhanced efficacy for seizure suppression [4, 5]. However, the preclinical and clinical evidence for optimal combinations is still sparse and no definitive evidence-based indications can be reached to guide clinicians in this choice [6]. The goal of “rational polytherapy” has not yet been achieved [5, 7].

Clinical trials designed to test the validity of the concept of rational polytherapy are difficult to perform. Nevertheless, clinical studies indicate a benefit from the addition of one or more AEDs [8, 9]. The combination of lamotrigine and valproate has been suggested to be particularly efficacious [4, 8, 10]. In this study we have used an *in vitro* seizure model to examine the seizure suppressive effects of three commonly used AEDs singly and in combination. This methodology allowed quantitative estimation of seizure inhibition without the potential pharmacokinetic complication of *in vivo* systems [11].

## Materials and Methods

### Ethics statement

This study was carried out in strict accordance with the Australian Code of Practice for the care and use of animals for scientific purposes. This study and all surgical procedures on rats were approved by the University of Melbourne Animal Ethics Committee (ethics numbers 1212450 and 15-057-UM). The rats were anesthetized with Lethabarb via intraperitoneal injection before sacrificing (decapitation) and all efforts were made to minimize suffering of the animals.

### Slice preparation

Hippocampal slices were prepared from non-epileptic control Wistar rats bred and purchased from the University of Melbourne Biological Resources Facilities (postnatal days 9–19, weights: 18.0–49.0 g, random genders of males and females, a total number of 45 rats used). The rats were first anesthetized with Lethabarb (325 mg/ml pentobarbitone sodium; approximately 3.5 mL per 1 kg body weight) via intraperitoneal injection and then decapitated. The brain was quickly removed, submerged in an ice-cold (0–1°C) cutting solution oxygenated with carbogen: 95% O_2_ + 5% CO_2_ before use and 450μm transverse hippocampal slices were sectioned with a vibratome (Zeiss—Microm, CSB 434, HM 650 V). The cutting solution was composed of (in mM): 87 NaCl, 2.5 KCl, 0.5 CaCl_2_.2H_2_O, 7 MgCl_2_.6H_2_O, 25 NaHCO_3_, 1.25 NaH_2_PO_4_, 25 D-glucose, and 75 sucrose.

The cut slices were then transferred to a holding chamber with a bicarbonate buffered saline (BBS) solution bubbled continuously with carbogen at room temperature (20–22°C) and were used after 40 minutes in this chamber. The holding BBS solution was composed of (in mM): 125 NaCl, 2.5 KCl, 2 CaCl_2_.2H_2_O, 1 MgCl_2_.6H_2_O, 25 NaHCO_3_, 1.25 NaH_2_PO_4_, and 25 D-glucose.

### Electrophysiological recordings

Individual hippocampal slices were transferred into a submerged slice recording chamber, mechanically stabilised and superfused with recording solutions continuously saturated with carbogen. A high potassium recording solution for induction of epileptiform discharges (ED’s) in hippocampal slices was composed of (in mM): 125 NaCl, 8.5 KCl, 2 CaCl_2_.2H_2_O, 1 MgCl_2_.6H_2_O, 25 NaHCO_3_, 1.25 NaH_2_PO_4_, and 10 D-glucose. Single and dual extracellular recordings were performed in the stratum pyramidale of CA1 and CA3 regions, using 3–5 MΩ micropipettes pulled from thin walled 1.5 mm diameter borosilicate glass capillaries (Sutter Instrument, SDR, Clinical Technology).

The glass microelectrodes were filled with the recording solution, and positioned at a depth of about ¼ to ½ of the slice thickness, which optimised recording quality of ED’s (usually with spike events between 1.0–3.0 mV amplitude and 1–9 Hz frequency). Positioning of the recording electrodes began 5–6 minutes after the transferred slice superfused with the high-K^+^ solution. The extracellular signals were recorded in current clamp mode with a MultiClamp 700B amplifier, Axon CNS, Molecular Devices; an analog-to-digital converter, NI USB-6259 (BNC); WinWCP software (Strathclyde Electrophysiology Software) was used for recording and Clampfit 10.2 (Molecular Devices) used for analysis. The data sampling interval was 100μs.

### Pharmacological testing

Drugs: lamotrigine, phenytoin (acid) and valproate (sodium salt) were purchased from Octagon Chemicals Limited, Hangzhou, China, Sigma-Aldrich, Life Science and Sapphire Bioscience, Cayman Chemical Company, respectively. The drugs were prepared as stock solutions dissolved in solvents: dimethyl sulfoxide (DMSO) for LTG and PHT; and Milli-Q water for VPA, stored at -20°C. On the day of experimentation, thawed drug solutions were diluted into recording solutions. The solvent DMSO was diluted to a final concentration of ≤ 0.1% DMSO—this concentration of DMSO had no effect on ED’s.

To assess combination effects, all drugs were applied to the slice by superfusion at a rate of ~2 ml/min with a pump from NanBei, ZhengZhou Nanbei Instrument Equipment Co., Ltd.; the temperature was set at ~32°C with a Warner TC-324B temperature controller, using the following protocol:

$$Baseline\;\left[Drug^{1}\right]\;\left[Drug^{2}\right]\;\left[Drug^{1}\right]+\left[Drug^{2}\right]\;Washout$$

The slice was bathed with each drug applications and washout (WO) for 15 minutes and 18 minutes, respectively. Spike discharge frequency stabilised typically after 10 minutes. Spike events under drug effects and WO were measured in the last 2 minutes of the 15-minute and 18-minute recording periods. The spikes were counted semiautomatically using a threshold search algorithm in Clampfit. The number of spike events under drug and WO effects were calculated as percentage (%) reduction relative to the baseline. The data from CA1 and CA3 regions of stratum pyramidale were combined for quantitative analyses to capture broad drug effects. There was no significant difference of drug effects between CA1 and CA3 areas.

### Statistical analyses for the drug effects

Percentage of spike events relative to baseline was expressed as mean ± standard error of the mean (SEM) of “n” recordings; error bars also indicate SEM. D'Agostino & Pearson omnibus normality test (alpha = 0.05) was performed prior to further statistical tests. Comparison among multiple relevant groups was performed through the one-way ANOVA test followed by a *post hoc* test using GraphPad Prism, to determine significant differences among the means of the relevant groups. Student’s t-test was used to compare the predicted additive effect (if individual sample sizes were different, the variance sum law of two independent random variables was used to calculate SEM of the predicted additive mean) and the actual combination response (see also [12]). The statistical significance of differences was assessed with a significance level set at p-value <0.05.

## Results

### 1. Characteristics of the high-K^+^ (8.5mM) seizure model

**Fig 1:**
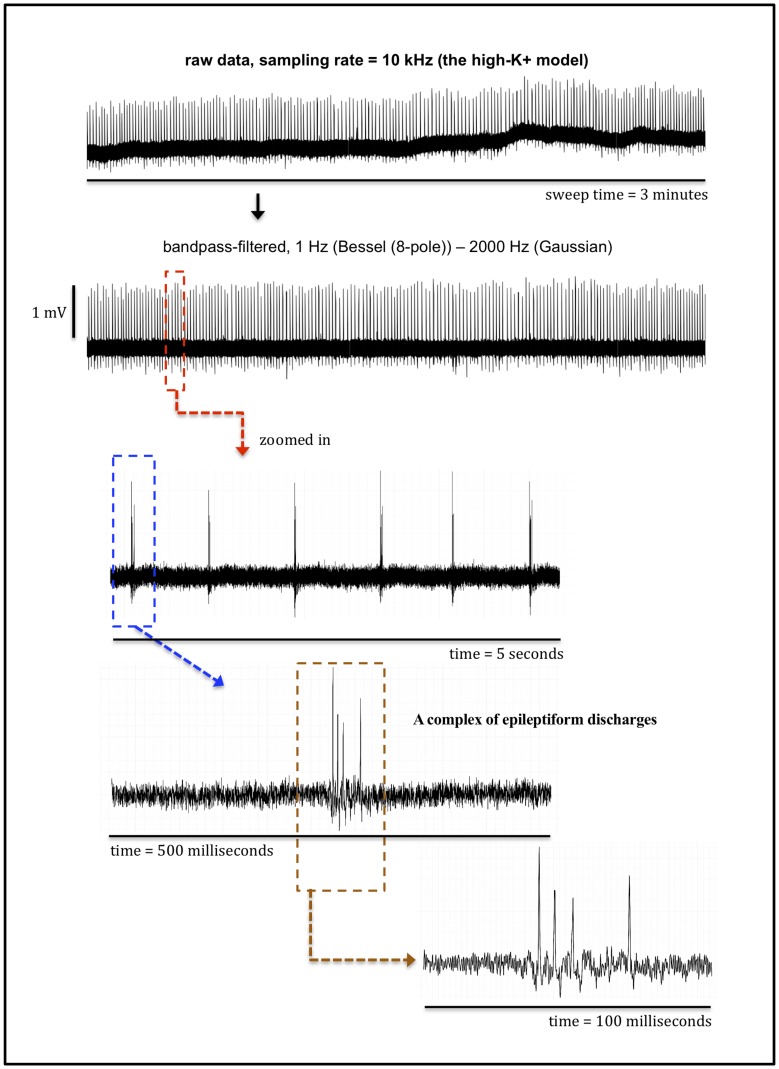
Example of epileptiform discharges observed in the stratum pyramidale of the high-K^+^ treated hippocampal slices.

The high-K^+^ model displayed a tonic-firing pattern with spike discharges with similar amplitudes and relatively stable frequencies (Fig 1).

These spikes appeared about 6 minutes after the superfusion of high-K^+^ solution. The repetitive spike discharges most likely resulted from multiple effects of the increased extracellular K^+^ concentration such as effective reduction of K^+^ conductances resulting in depolarization of neuronal cell membranes [13, 14], interference with Cl^−^extrusion leading to a depolarizing shift of chloride equilibrium potential, and increases in transmitter release [15].

### 2. PHT-VPA combination produced additivity of SLD suppression

**Fig 2:**
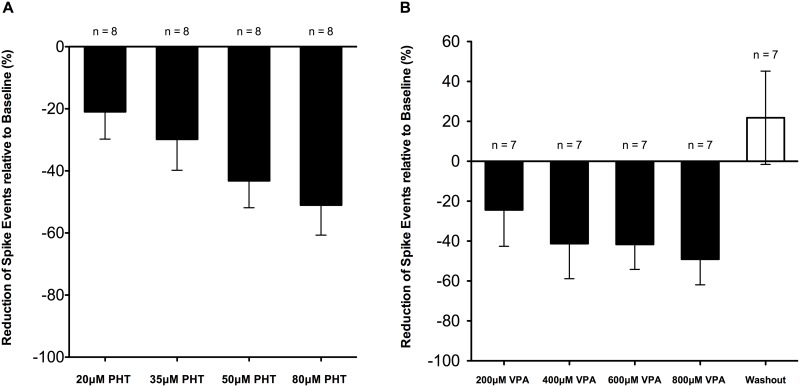
Dose responses of PHT and VPA in suppressing population spike events in the high-K^+^ model. Data from combined recordings of CA1 and CA3 regions were expressed as relative percentage (%) reduction compared to the baseline. (**A**) The effect of PHT on spike frequency was taken during the final 1-minute interval following 3-minute drug applications. (**B**) The effect of VPA on spike frequency was taken over the stable final 2-minute interval following 6-minute applications of VPA and washout. All data were shown as mean ± SEM; “n” indicates the number of recordings.

In initial experiments, dose-response profiles of PHT and VPA were characterised. PHT took about 3 minutes for full effect and produced 21.0 ± 8.8%, 29.8 ± 10.0%, 43.2 ± 8.6%, 51.0 ± 9.6% inhibition of spike events at 20 μM, 35 μM, 50 μM, and 80 μM PHT, respectively (n = 8, from 5 slices) (Fig 2A). Although SLD suppression was dose-dependent with PHT, complete spike suppression was not achieved with PHT concentrations within the human therapeutic range of blood plasma concentrations [16, 17]. VPA spike suppression typically stabilised after about 6 minutes and produced 24.4 ± 18.2%, 41.3 ± 17.6%, 41.7 ± 12.5%, 49.1 ± 12.8% inhibition of spike events at 200 μM, 400 μM, 600 μM, and 800 μM VPA, respectively (n = 7, 5 slices) (Fig 2B). Although there was a trend to increasing suppression of discharges with higher concentrations of VPA, this did not quite reach statistical significance, probably because of the small sample size and inter-trial variability. Washout resulted in not only an increase in spike frequency, and interestingly an actual increase in SLD frequency of 21.8 ± 23.4% (n = 7) (Fig 2B). Overall, spike discharges in this model were strongly suppressed, but not completely eliminated by PHT and VPA applied singly at clinically relevant concentrations [16, 17].

**Fig 3:**
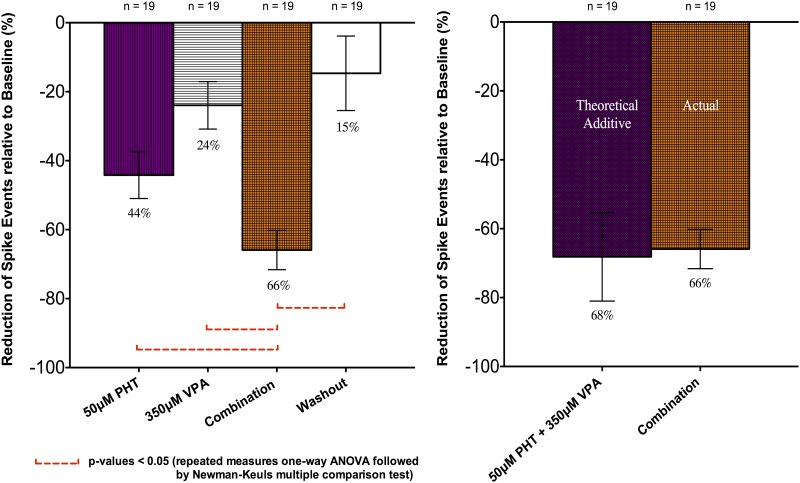
Effects of PHT, VPA applied singly and in combination upon frequency of spike events. Data from combined recordings of CA1 and CA3 regions were expressed as relative percentage (%) reduction compared to the baseline. All data were shown as mean ± SEM; “n” indicates the number of recordings.

**Fig 4:**
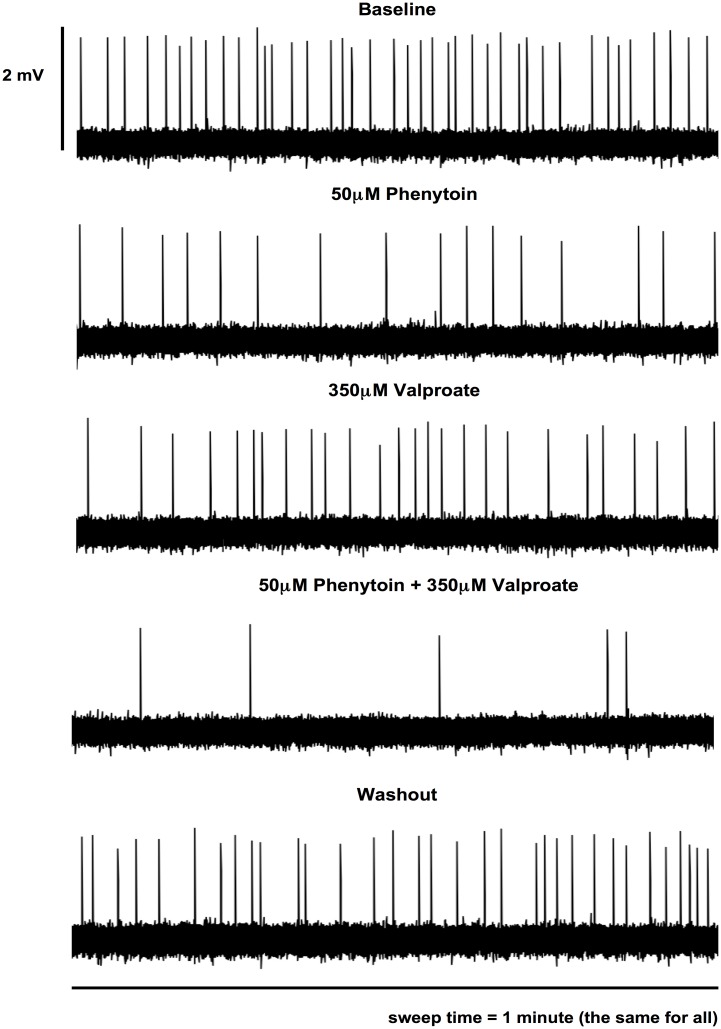
Representative traces of effects of AEDs (PHT, VPA) and washout upon neuronal population spike events. Spike events over 1-minute intervals were from one continuous recording during baseline, drug applications and washout, for qualitative displays.

Based on the dose responses of PHT and VPA described above, 50μM PHT and 350μM VPA were chosen to examine combination effects. The combination of PHT and VPA had an “additive” effect in that the total percentage suppression of spike discharges was close to the sum of the individual drug suppression effects (Figs 3 and 4). In particular, 50μM PHT produced 44.2 ± 6.8%, and 350μM VPA 23.9 ± 6.9% spike discharge suppression, with 65.9 ± 5.7% inhibition with the combination (n = 19, 17 slices). This value did not differ significantly from a predicted percentage inhibition of 68.2 ± 12.9% calculated by simply adding the individual effects. The combination effect was also significantly different from the individual effects of 50μM PHT and 350μM VPA with p-values < 0.05 (one-way ANOVA followed by Newman-Keuls Multiple Comparison Test). In summary, these experiments showed that combination of two drugs used effectively in combination in clinical practice, was characterised by a linearly additive suppression of epileptiform discharges (Fig 3).

Washout after combined drug combination resulted in an increase in discharge firing rate, mostly with some residual inhibition (14.7 ± 10.8%; n = 19; Figs 3 and 4) compared to baseline. However, there was a clear difference between the combination effect and washout. The increase in spike rate after washout suggested that the drug effects were not due to time-dependent run down.

**Fig 5:**
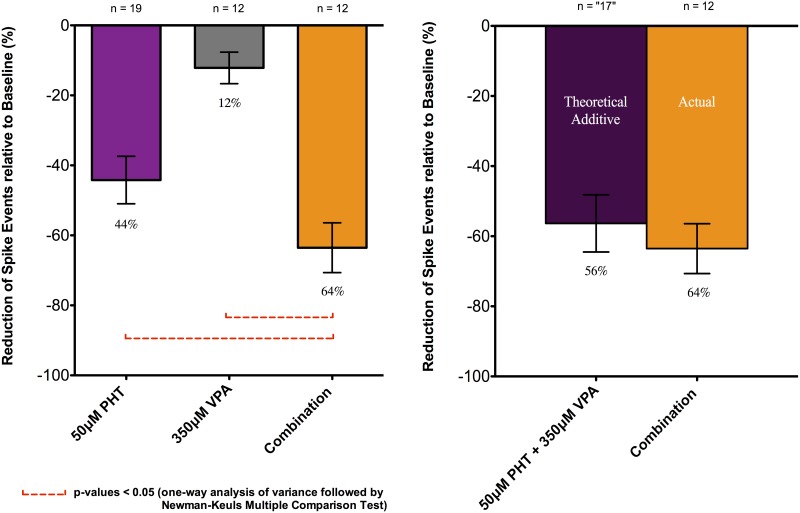
Effects of PHT, VPA applied in isolation and in combination upon frequency of spike events. Data were expressed as relative percentage (%) reduction compared to the baseline. All data were shown as mean ± SEM; “n” indicates the number of recordings from different slices. See text for details.

As the protocol above used several drug treatments in the same sequence over time had potential confounders including repeated measures errors and long duration of observation, we sought to validate these observations with a simplified, more focused set of observations using different slices for each drug applications over shorter periods of time. 350μM VPA and the combination of 350μM VPA and 50μM PHT were independently re-assessed on different slices with the same individual testing periods and analyses—spike events under drug effects were counted in the last 2 minutes of the 15-minute recording period and were calculated as percentage reduction relative to baseline. The drug effects again summed linearly (Fig 5). 350μM VPA alone and the combination of 350μM VPA and 50μM PHT produced 12.2 ± 4.5% (n = 12, 12 slices) and 63.5 ± 7.1% (n = 12, 12 slices) inhibition of spike events, respectively. From previous experiments (Fig 3), 50μM PHT produced 44.2 ± 6.8% (n = 19, 17 slices) and this value was used for comparison. There were statistically significant differences between the combination effect compared to the 350μM VPA, and to the 50μM PHT with p-values < 0.05 (one-way ANOVA followed by Newman-Keuls Multiple Comparison Test). A predicted additive suppressive effect between 350μM VPA and 50μM PHT was calculated to be 56.3 ± 8.2% (the variance sum law of two independent random variables was used to calculate SEM of the predicted additive mean and the corresponding sample size rounding to the nearest whole number of “17” was also derived). However, there was no significant difference between the predicted additive (56.3 ± 8.2%) and the actual combination effect (63.5 ± 7.1%) (Fig 5).

### 3. LTG-VPA combination produced supra-additivity of SLD suppression

**Fig 6:**
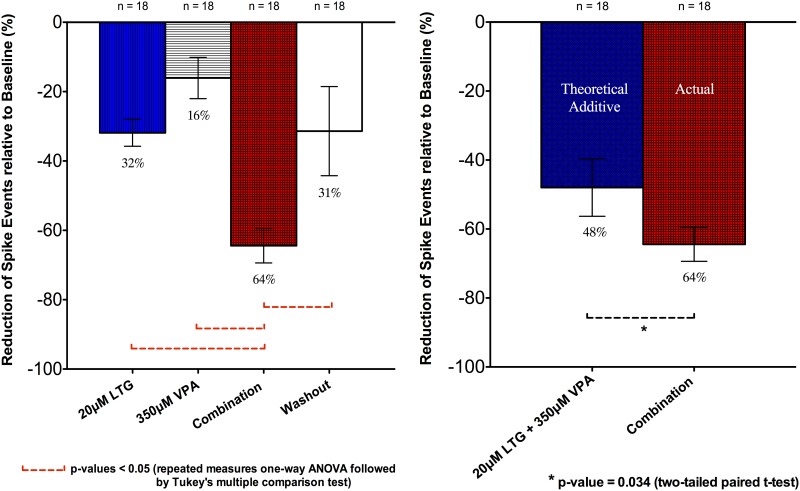
Effects of LTG, VPA applied singly and in combination upon frequency of spike events. Data from combined recordings of CA1 and CA3 regions were expressed as relative percentage (%) reduction compared to the baseline. All data were shown as mean ± SEM; “n” indicates the number of recordings.

**Fig 7:**
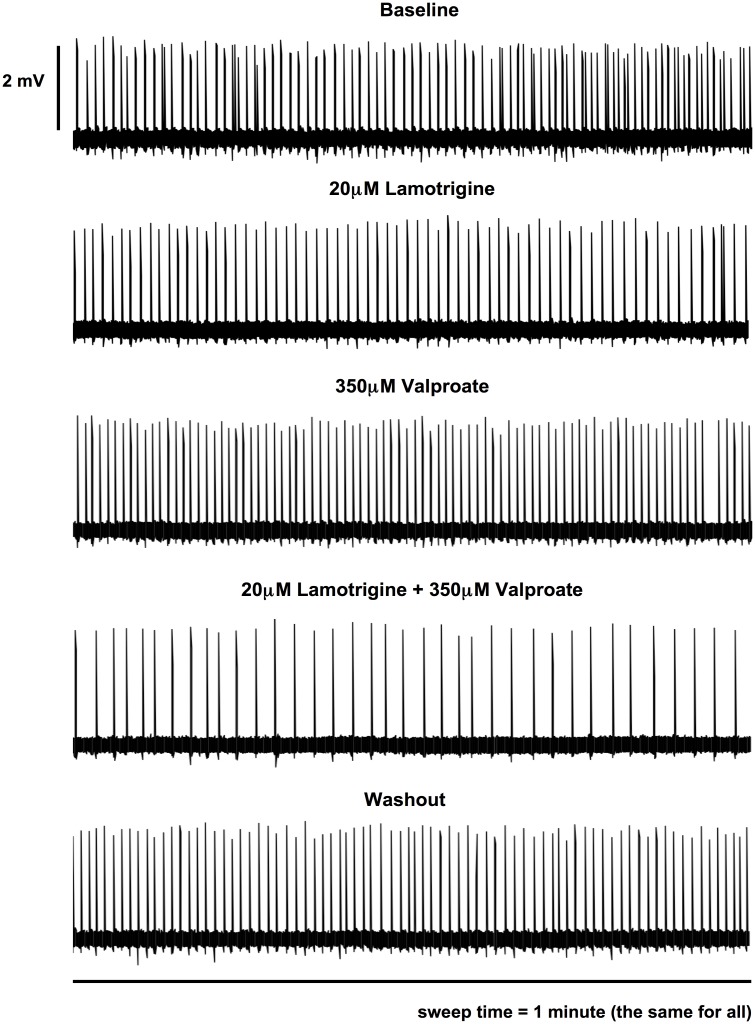
Representative traces of effects of AEDs (LTG, VPA) and washout upon neuronal population spike events. Representative 1-minute intervals of spike events during baseline, drug applications and washout from one continuous recording are displayed.

The drug combination of LTG and VPA was next tested. This was of particular interest as this is considered to be particularly efficacious in clinical practice for both focal and generalised seizures [4, 8, 10]. The spike suppressive effects of different concentrations of LTG (5μM, 10μM, 20μM) were measured initially and 20μM LTG, the upper limit of the human therapeutic plasma range [16], reduced spike frequency by about one third. This 20 uM concentration was then tested with 350μM VPA in combination. A clear trend towards supra-additive effect was noted (Figs 6 and 7). Individual drug application of 20μM LTG and 350μM VPA produced reductions of 31.9 ± 3.9% and 16.1 ± 5.9%, respectively (n = 18; 12 slices). The combined drug concentrations produced 64.4 ± 5.0% SLD suppression (n = 18; 12 slices) significantly greater than the individual 20μM LTG and 350μM VPA applications with p-values < 0.05 (one-way ANOVA followed by Tukey’s Multiple Comparison Test). As compared to the predicted sum of individual effect: 48.0 ± 8.3% (linear summation), the degree of suppression by the actual combination (64.4 ± 5.0%) was also significantly greater with a p-value of 0.034 (two-tail paired t-test) (Fig 6). These observations thus disclosed supra-additivity of effect with the combination of LTG and VPA.

Washout produced notable increase in SLD frequency, though as with the phenytoin/valproate combination did not generally return to baseline values; the remaining “inhibition” was 31.4 ± 12.8% (n = 18; 12 slices). However, washout was significantly different from the combination effect with a p-value of 0.05 (one-way ANOVA followed by Tukey’s Multiple Comparison Test). Interestingly, washout after this combination of LTG and VPA seemed to be less effective than washout after the combination of PHT and VPA (Fig 3) with the same testing protocol.

**Fig 8:**
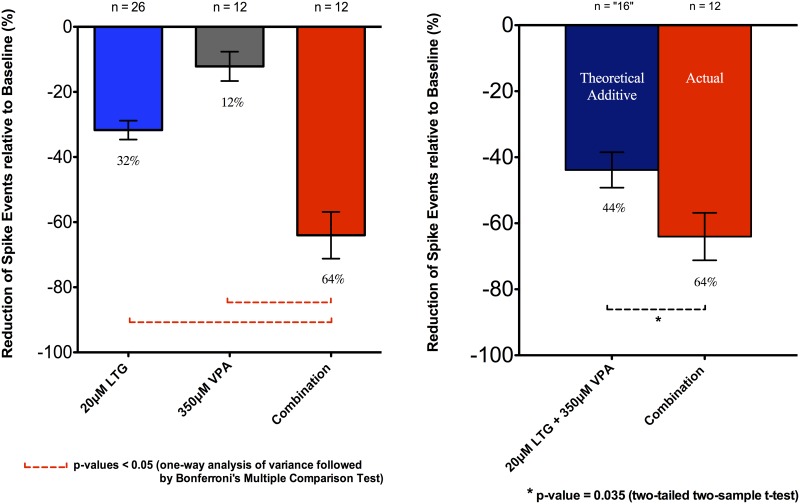
Effects of LTG, VPA applied in isolation and in combination upon frequency of spike events. Data were expressed as relative percentage (%) reduction compared to the baseline. All data were shown as mean ± SEM; “n” indicates the number of recordings from different slices. See text for details.

Further observations using the simplified protocol (using different slices for each drug applications) were also made to corroborate results obtained from the above experiment protocol (Fig 8). The combination of 20μM LTG and 350μM VPA was further independently tested on different slices with 64.0 ± 7.2% (n = 12, 12 slices) inhibition of spike events in this second set of experiments. This effect was then compared (unpaired t-test) with the individual drug effects previously tested above, i.e., 350μM VPA with 12.2 ± 4.5% (n = 12, 12 slices), and 20μM LTG (with extra recordings) with 31.7 ± 2.9% (n = 26, 17 slices). The drug combination again summed supra-linearly. There were statistically significant differences between the combination effect compared to the 350μM VPA, and to the 20μM LTG with p-values < 0.05 (one-way ANOVA followed by Bonferroni’s Multiple Comparison Test) (Fig 8). A predicted additive suppressive effect between 350μM VPA and 20μM LTG was calculated to be 43.9 ± 5.4%, with the standard error derived by the variance sum law of two independent random variables. The difference between the predicted additive (43.9 ± 5.4%) and the actual combination effect (64.0 ± 7.2%) was significant with a p-value of 0.035 (two-tailed two-sample t-test) (Fig 8).

## Discussion

### The high-K^+^ seizure model

Superfusion of the high-K^+^ (8.5 mM) external solution is known to induce epileptiform discharges (ED’s) in the stratum pyramidale of rat hippocampal slices, and this preparation has been used previously to test drug effects [18, 19]. This produced a consistent tonic-firing pattern of spike events that in this study allowed reliable quantification of AED effects (see also [18]). While the precise correlation of this model with human epilepsy is not completely clear, it does have characteristics comparable to human epileptic discharges, for example, ‘periodic epileptiform discharges’ [20–26]. The high-K^+^ preparation may also correspond to the increased potassium levels in the extracellular microenvironment during *in vivo* seizures, and thus, may be a useful model of focal epileptiform activity [15, 27]. A notable advantage of this preparation is that commonly used AEDs with clinically relevant concentrations have significant suppressive effects on ED’s (see also [28–31]), again suggesting that it is a useful technique for assessing AED effects.

### Combination efficacy of AEDs: PHT and VPA, LTG and VPA

Epileptic seizures are likely to result from a combination of complex pathological factors at cellular and molecular levels, including excitatory-inhibitory imbalance [32], changes in extracellular ionic concentrations [27], dysfunctional plasticity changes, resulting in failure of inhibition of neuronal hyper-excitability [33]. Such multiple mechanisms may help to explain why seizure control is frequently difficult to achieve with AED monotherapy. For that reason, treatment with combinations of AEDs with different mechanisms of action has been used in an attempt to provide better seizure control in epilepsy patients [34, 35], although there has been little strong evidence of the effectiveness of this approach. In other areas of medicine, the use of multiple drugs that act on different targets has also been successfully employed, resulting in significant improvements in treatment efficacy of conditions such as HIV [36], Hepatitis C infection [37], and cancer [38, 39], thus providing a paradigmatic precedent for this strategy.

In this *in vitro* study, we examined two combinations of AEDs, LTG and VPA, PHT and VPA. LTG has several known effects, including modulating voltage-dependent sodium channels to limit repetitive firing of neurons, decreasing voltage-gated calcium currents and inhibiting post-synaptic AMPA receptors, all potentially contributing to a decrease in neuronal excitability [40–43]. PHT is a known sodium channel modulator, targeting the inactivated state, reducing repetitive firing of sodium ion-dependent action potentials [44–46]. VPA has several incompletely understood mechanisms of action [47], but its anti-epileptic effect has been linked to potentiation of GABAergic transmission and weak blockade of voltage-dependent sodium ion channels [45, 48–51].

We observed about 44% and 24% reduction of discharge frequency with 50uM PHT and 350uM VPA, respectively (Fig 3). However, if the two drugs were added together, then three possible effects [34, 52] might be predicted:

1. spike inhibition significantly lower than the predicted additive (68%) would indicate **sub-additivity** or **antagonism**
2. spike inhibition not significantly different from the predicted additive (68%) would reflect a trend towards linear **additivity**
3. spike inhibition significantly greater than the predicted additive (68%) would imply **supra-additivity** or **synergy**.

These three scenarios provide a relative framework to assess drug combination effect. Notably, a ‘constant relative potency ratio’ based on the dose equivalence concept (see [53]) did not have to be assumed for the two active drugs as is necessary for isobolographic analysis using animal models [54, 55] (see also [56] for review). Interestingly, our dose-response data was not consistent with the constant relative potency ratio.

Additivity of effect was observed in combinations of PHT and VPA (Figs 3 and 5). This linear additivity of effect is in itself quite intriguing, suggesting that the two drugs might be expected to be more efficacious in combination, as has been found clinically [4].

The combination of 20uM LTG and 350uM VPA produced a supralinear “synergistic” effect (Figs 6 and 8) in contrast to the additive suppressive effect of PHT and VPA. This supra-additivity of effect correlates with possible supra-additive anti-convulsant effects inferred from clinical studies [8] as well as preclinical data from an animal model using isobolographic analysis [54].

The mechanisms of synergistic interaction are not clear; the observation of the additive effect of phenytoin with valproate contrasts with the synergistic interaction with lamotrigine and suggests a difference in action between these two drugs both of which are known to significantly reduce the amplitude of sodium current, with a presumed common binding site [44]. It is possible that an additional anti-glutaminergic effect of LTG [42] may be the basis for the synergistic interaction.

The effects of washout of drug combinations were also intriguing (Figs 3 and 6); in general there was a strong recovery of firing, and in some cases, above baseline firing, which was unexpected. However, recovery was not generally complete and this may relate to non-specific run down of the preparation, or due to the lipophilic drugs sequestered in the brain slice. Another interesting possibility is that there may have been some longer term reduction in excitability, which could be a focus of future experiments.

A limitation of the current study is the observation of only a single combination of drug concentrations for the combination studies. While it is unlikely that the differences in efficacy of the combinations are a dosage effect, it would be useful to explore a wider range of drug concentrations, looking for optimal concentration ratios [57].

## Conclusions

These experiments have shown both additive and supra-additive interactions in suppression of epileptiform events in the hippocampal slice, correlating with clinical observations of drug combination efficacy [4, 8]. This methodology should allow further exploration of these mechanisms, effects on synaptic transmission, voltage gated conductances and network level effects.

Furthermore, this *in vitro* estimative method may also provide a drug assay to identify potentially efficacious drug combinations (ethosuximide and valproate [6], lacosamide and levetiracetam [55], levetiracetam and topiramate [58]) for further mechanistic and clinical research.
